# Malignant Transformation of Giant Cell Tumor of Bone and the Association with Denosumab Treatment: A Radiology and Pathology Perspective

**DOI:** 10.1155/2022/3425221

**Published:** 2022-06-17

**Authors:** K. van Langevelde, A. H. G. Cleven, A. Navas Cañete, L. van der Heijden, M. A. J. van de Sande, H. Gelderblom, J. V. M. G. Bovée

**Affiliations:** ^1^Department of Radiology, Leiden University Medical Center, Leiden, Netherlands; ^2^Department of Pathology, Leiden University Medical Center, Leiden, Netherlands; ^3^Department of Pathology, University Medical Center Groningen, Groningen, Netherlands; ^4^Department of Orthopedics, Leiden University Medical Center, Leiden, Netherlands; ^5^Department of Medical Oncology, Leiden University Medical Center, Leiden, Netherlands

## Abstract

**Objective:**

Malignancy in giant cell tumor of bone (mGCTB) is categorized as primary (concomitantly with conventional GCTB) or secondary (after radiotherapy or other treatment). Denosumab therapy has been suggested to play a role in the etiology of secondary mGCTB. In this case series from a tertiary referral sarcoma center, we aimed to find distinctive features for malignant transformation in GCTB on different imaging modalities. Furthermore, we assessed the duration of denosumab treatment and lag time to the development of malignancy.

**Methods:**

From a histopathology database search, 6 patients were pathologically confirmed as having initial conventional GCTB and subsequently with secondary mGCTB.

**Results:**

At the time of mGCTB diagnosis, 2 cases were treated with denosumab only, 2 with denosumab and surgery, 1 with multiple curettages and radiotherapy, and 1 with surgery only. In the 4 denosumab treated patients, the mean lag time to malignant transformation was 7 months (range 2–11 months). Imaging findings suspicious of malignant transformation related to denosumab therapy are the absence of fibro-osseous matrix formation and absent neocortex formation on CT, and stable or even increased size of the soft tissue component.

**Conclusion:**

In 4 patients treated with denosumab, secondary mGCTB occurred within the first year after initiation of treatment. Radiotherapy-associated mGCTB has a longer lag time than denosumab-associated mGCTB. Close clinical and imaging follow-up during the first months of denosumab therapy is key, as mGCTB tends to have rapid aggressive behavior, similar to other high-grade sarcomas. Nonresponders should be (re) evaluated for their primary diagnosis of conventional GCTB.

## 1. Introduction

Giant cell tumor of bone (GCTB) typically occurs in young adults between 20 and 40 years of age after closure of the physis. GCTB arises from the epi-metaphysis and extends up to the subchondral bone plate [1]. The 2020 WHO classification of soft tissue and bone tumors [2] defines GCTB as a locally aggressive tumor that rarely metastasizes. A further division is made into two subtypes: conventional and malignant GCTB. Conventional GCTB contains three cell types: neoplastic mononuclear stromal cells, macrophages, and osteoclast-like giant cells. The neoplastic mononuclear stromal cells express a receptor activator of nuclear factor kappa-B ligand (RANKL) which binds with the RANK receptor on osteoclast precursors. Via the RANK-RANKL signaling pathway these cells induce osteoclast formation which gives the typical osteolytic appearance to the tumor [3]. The incidence of GCTB is 1.66 per million inhabitants per year, based on a nationwide pathology database study in the Netherlands. During the 5-year study period, from January 2009 to December 2013, a total of 138 new cases of GCTB were found, with only 1 case of malignant GCTB [1].

H3F3A gene mutations are detected in at least 95% of giant cell tumors, and 90% of these mutations are represented by the H3.3 pGly34Trp mutation [2]. H3.3 pGly34Trp (H3G34W) immunohistochemistry is a reliable surrogate marker for molecular analysis [4]. Within the context of bone tumors, this marker is highly specific for GCTB.

The primary treatment for GCTB is surgical excision. The rate of local recurrence depends on the type of surgery performed and ranges from 10 to 50% for curettage with local adjuvants and 5% for wide resection [5–9]. Medical treatment for GCTB includes denosumab, a monoclonal antibody which binds to RANKL and inhibits bone destruction and osteolysis [3]. Histologically, denosumab induces intralesional deposition of bone with a strong depletion of giant cells. Early after treatment, the cellularity is high, with haphazard bone deposition, while after prolonged therapy cellularity decreases while the new bone is deposited as broad, rounded cords or long, curvilinear arrays [10]. Thus, the morphology after treatment is variable and can resemble osteosarcoma, though significant nuclear atypia, mitotic activity, and infiltration of preexisting bone are absent [10].

Neo-adjuvant treatment with denosumab is relevant in cases where surgical resection may result in severe morbidity (such as joint reconstruction or amputation) and a short-term treatment of three months is given to facilitate surgery and decrease tumor pain [11]. Long-term denosumab may be used as a primary treatment in patients who have an unresectable tumor (often in the spine or sacrum). Denosumab treatment has been shown to limit tumor progression, reduce tumor size, increase bone formation and bone mineral density, reduce pain, and improve functional status [5, 6, 12, 13].

Malignancy in GCTB (mGCTB) is categorized by the WHO classification as primary (a nodule of highly pleomorphic, neoplastic mononuclear cells occurring concomitantly with an otherwise conventional GCTB) or secondary (occurring after treatment often involving radiotherapy) [2]. In secondary malignant transformation, the conventional GCTB may or may not be detectable [2, 14–16]. The malignant component does not have specific histological features and may be either an undifferentiated sarcoma or an osteosarcoma with telangiectatic or osteoblastic features [2, 8, 16]. Tahir et al. recently reviewed the literature on mGCTB and found that in secondary mGCTB, morphological subtypes were osteosarcoma in 58%, fibrosarcoma in 32%, and undifferentiated pleomorphic sarcoma in 10% (*N* = 84 cases) [17]. In mGCTB, the H3G34W mutation can be either retained or absent [2, 18–20]. Strong expression of p53 has been described in a subset of secondary mGCTB [2].

There is an ongoing debate about denosumab being a risk factor for mGCTB. The largest prospective clinical trial to date (*n* = 532) reported 1% of confirmed sarcomatous transformation in GCTB patients on denosumab [13]. Several cases of sarcomatous transformation in *recurrent* GCTB have been described, respectively, in the tibia with transformation into a high-grade pleomorphic sarcoma after 13 months on denosumab treatment [21], and in the ischium with transformation into a high-grade osteosarcoma after 6 months on treatment [18]. However, whether sarcomatous transformation in (recurrent) GCTB is a causal or coincidental phenomenon with regards to the use of denosumab is unclear for these cases, and as yet no biological hypothesis exists that explains the association between denosumab treatment and malignant transformation.

In this case series, we aim to give an overview of mGCTB patients from our tertiary referral center (Leiden University Medical Center). We aim to find distinctive imaging features for malignant transformation in GCTB on different modalities. In addition, we will assess the duration of denosumab treatment as a possible risk factor for secondary malignant transformation and review lag time to the development of malignancy.

## 2. Methods

The LUMC pathology database was searched for the diagnosis of GCTB with atypical features and mGCTB using diagnostic codes. 17 potential cases were found and a review of cases was performed both radiologically (by two MSK oncology radiologists, KvL and ANC) and histologically (by two bone and soft tissue tumor pathologists, AHGC and JVMGB). A clinical oncologist (HG) reviewed the medical history involving denosumab as to obtain the duration of treatment and time interval before diagnosis of malignant transformation (lag time). An orthopaedic surgeon (LvdH) reviewed the medical charts for symptoms of pain or new functional impairment preceding malignant transformation. All samples were handled according to the ethical guidelines described in the “Code for Proper Secondary Use of Human Tissue in the Netherlands,” as approved by the Leiden University Medical Centre ethical board. Informed consent was obtained from the subjects (through the Dutch Bone Tumor Committee and LUMC biobank) or, in case they were deceased, the next of kin gave consent. Molecular analysis (H3G34W immuno or targeted NGS) was performed on biopsy samples and resection specimens before and after malignant transformation depending on the availability of samples.

As for radiological assessment of the cases, conventional images (X-ray), computed tomography (CT), ^18^F-FDG-positron emission tomography-CT (PET-CT) and magnetic resonance imaging (MRI) were taken into account depending on the availability in our LUMC PACS (some patients were referred from elsewhere). Radiological features of malignant GCTB were described as we aimed to find distinctive imaging features for malignant transformation. All cases were discussed during multidisciplinary consensus meetings.

Cases were categorized as primary or secondary mGCTB according to the WHO 2020 criteria: primary mGCTB is composed of a nodule of sarcomatous growth juxtaposed to zones of conventional GCTB, and secondary mGCTB is a sarcomatous growth that occurs at the site of a previously documented benign GCTB after treatment [2, 14, 22]. Subgroups of secondary malignant GCTB were made based on risk factors such as surgery, radiotherapy, and denosumab or a combination of two or more of these risk factors. The criteria used to distinguish mGCTB from conventional denosumab related treatment changes in GCTB included nuclear atypia (especially hyperchromasia), high mitotic activity, atypical mitotic figures, extensive necrosis, and infiltration of preexisting bone [2, 10]. Of note, we did not include cases of other bone sarcomas harbouring a H3G34W mutation into this case series, if they were histologically not related to GCTB, as it is at present unclear whether these represent malignant GCTB [2].

## 3. Results

Of the 17 potential cases, six were defined as mGCTB after pathology review. An overview of the cases is given in Table 1. The mean age was 42 years (range 27–55) at the time of GCTB diagnosis. Five out of six patients (83%) were female. The tumor sites were distal femur (*n* = 2), tarsal navicular (*n* = 1), ilium (*n* = 1), sacrum (*n* = 1), and lumbar spine (*n* = 1).

All cases were secondary mGCTB. Before the diagnosis of mGCTB was confirmed, 4 out of 6 patients reported an increase in pain and functional impairment; 2 underwent their routinely scheduled follow-up imaging and in 2 patients imaging was performed earlier than their regularly planned outpatient visits due to clinical complaints. At the time of mGCTB diagnosis, two patients were treated with denosumab only, two with denosumab and surgery, one with multiple curettages due to recurrence followed by radiotherapy, and one case was treated with surgery only. In all four denosumab treated patients, the mean lag time between the start of denosumab treatment and malignant transformation was 7 months (range 2–11 months). In the subgroup treated with denosumab only (*n* = 2), the mean lag time was 6.5 months (range 2–11 months). In the two patients treated with both surgery and denosumab, the mean lag time was 7.5 months (range 5–10 months). The case with radiation-associated malignant GCTB had a lag time of 13 years. The patient treated with surgery only had a latency period of 9 months between surgery and mGCTB diagnosis. The clinical outcome is shown in Table 1.

Only two out of six cases showed imaging findings at the primary tumor location suspicious of malignant transformation, even when reviewed retrospectively with histopathological guidance. Case 2 presented with a new tumor location, i.e., a metastasis elsewhere in the spine. Imaging features suspicious of malignant transformation were noted in cases 1 and 3. These cases are described in more detail below (and in Table 2). In two other cases, mGCTB was presented with findings similar to a local recurrence (cases 4 and 6). A mixed response to denosumab was noted in case 5 that showed bone deposition and neocortex formation following denosumab on CT, however no decrease in tumor size. Imaging characteristics pointing towards malignant transformation are grouped by modality in Table 3. For the musculoskeletal radiologist, key findings pointing towards malignant transformation are: absence of fibro-osseous matrix formation and neocortex formation on CT, and stable or increased size of the soft tissue component on CT or MRI while on denosumab treatment.

### 3.1. Case 1

**Figure 1:**
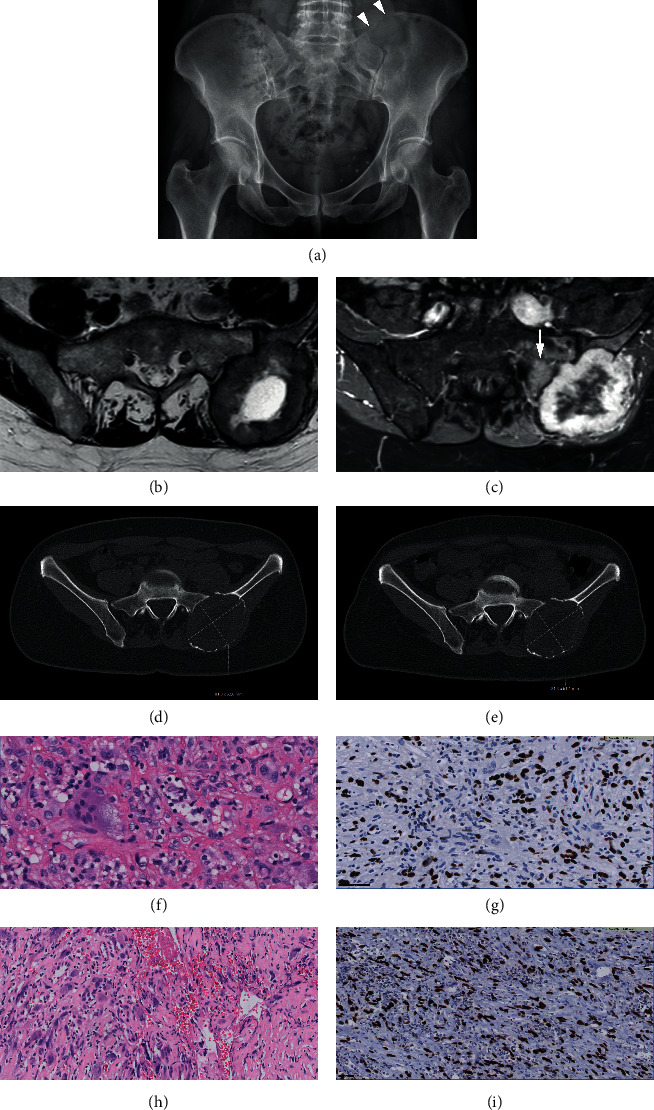
Case 1, a 54-year-old female with a GCTB in the left ilium. (a) X-ray at presentation shows an osteolytic tumor with cortical destruction (arrowheads) cranially in the left iliac wing, adjacent to the sacroiliac joint. (b) MRI at the time of diagnosis; axial T2 TSE shows an expansive tumor in the left posterior aspect of the ilium with central necrosis and a peripheral thick low signal intensity rim. (c) MRI at the time of diagnosis; T1 SPIR postgadolinium (Gd) shows heterogeneous enhancement, mainly of the tumor rim and adjacent bone marrow edema in the sacrum (arrow) and ilium. (d) Axial unenhanced CT images; 5 months after starting denosumab treatment and (e) 8 months after starting denosumab treatment. Both scans showed no decrease in size and no matrix formation centrally. No thick rim of neocortex was formed. (f) Histology; morphology of first biopsy confirmed the diagnosis of conventional GCTB: mononuclear stromal cells intermixed with osteoclast-like giant cells. The mononuclear cells have slightly enlarged nuclei and predominate over the giant cells, but since overt nuclear atypia and hyperchromasia and atypical mitoses are absent the diagnosis is still compatible with conventional giant cell tumor of bone. In the background reactive lymphocytes and some sclerosis. (g) Immunohistochemistry of the biopsy at the time of presentation: mononuclear stromal cells positive for H3G34W (scale bar 50 *μ*m). (h) Histology of resection after denosumab treatment showed malignant GCTB: atypical cells with enlarged hyperchromatic nuclei, scattered monstrous tumor cells, and atypical mitotic figures with matrix deposition suggestive of tumor osteoid. (i) Immunohistochemistry at the time of resection after denosumab treatment showed atypical stromal cells positive for H3G34W.

An X-ray at presentation showed an osteolytic tumor with cortical destruction in the left iliac wing (Figure 1(a)). Baseline MRI axial T2 TSE weighted sequence showed an expansive tumor in the left posterior aspect of the iliac wing with central necrosis and a peripheral thick low signal intensity rim (Figure 1(b)). T1 SPIR after contrast showed enhancement mainly of the tumor rim and, in addition enhancement of the adjacent bone marrow edema in the sacrum and ilium (Figure 1(c)). The diagnosis of conventional GCTB (H3G34W positive) was made after CT guided biopsy (Figures 1(f) and 1(g)), followed by the start of denosumab therapy. CT scans performed 5 and 8 months after starting denosumab treatment showed no decrease in tumor size and no increase tumor density (Figures 1(d) and 1(e)). Furthermore, no thick rim of neocortex was formed and multiple foci of cortical disruption persisted during treatment. Based on these worrisome radiological features, a resection was performed and the diagnosis of mGCTB was confirmed. Histology at the time of resection showed atypical cells with enlarged hyperchromatic nuclei, scattered monstrous tumor cells, atypical mitotic figures, and deposition of tumor osteoid. The stromal cells remained positive for H3G34W (Figures 1(h) and 1(i)).

### 3.2. Case 3

**Figure 2:**
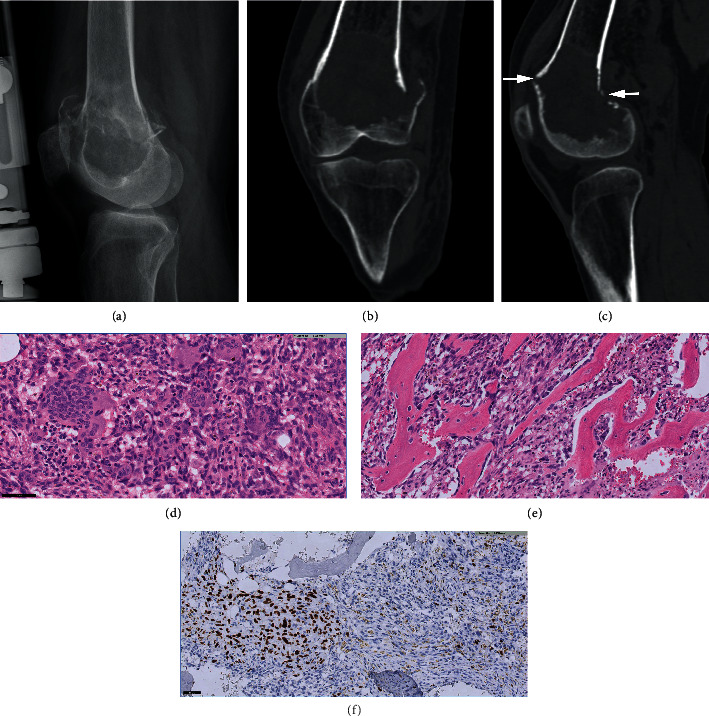
Case 3, a 55-year-old female with a pathological fracture of the femur with underlying GCTB. (a) X-ray at the time of diagnosis showed a pathological fracture through a well-defined osteolytic lesion with a sclerotic margin in the distal femur meta- and epiphysis, initially treated by external fixation. (b) Coronal and (c) sagittal CT images at the time of diagnosis showed the pathological fracture extending through the anterior and posterior cortices (arrows) and no internal matrix. (d) Morphology at the time of curettage shows many mononuclear stromal cells intermixed with large osteoclast-like giant cells without atypical morphological features. (e) At the time of curettage; areas with reactive woven bone with typical osteoblast lining are seen, which may be due to the clinical fracture. (f) At the time of curettage; heterogeneous positive H3G34W staining in stromal cells.

**Figure 3:**
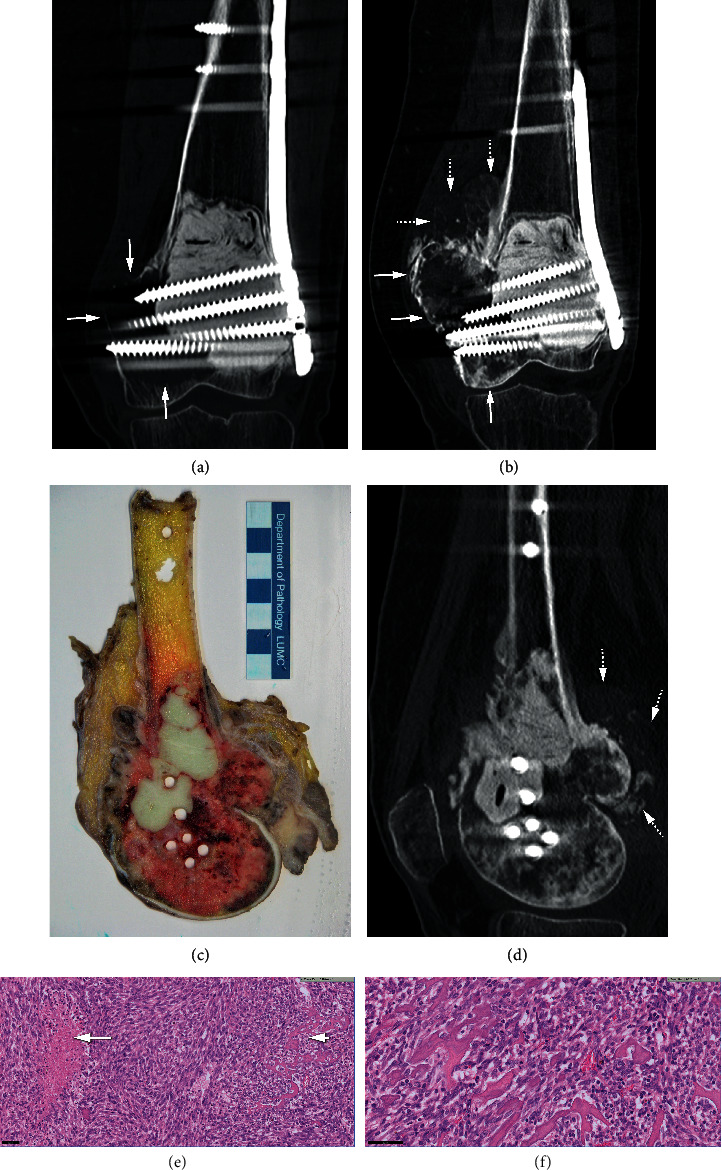
Case 3, same case as in Figure 2; local recurrence occurred 1 year after curettage, followed by denosumab treatment. After en bloc resection, the diagnosis of malignant GCTB was made. (a) Coronal CT image performed for follow-up approximately 1 year after surgery showed osteolysis along the medial bone-cement interface (arrows) in keeping with local recurrence. Denosumab treatment was started after this scan. (b) Coronal CT image after 10 months of denosumab therapy showed increased density in the osteolytic area of recurrence due to formation of fibro-osseous tissue (vertical arrow at the medial femoral condyle), the tumor expanded further into the soft tissues with an irregular margin (horizontal arrows). In addition, proximal to the area of local recurrence there was a newly formed component of osteoid matrix (dotted arrows), suspicious for progression to osteosarcoma. (c) Photograph of tumor macroscopy (sagittal section) after en bloc resection shows the cementum from the previous surgery, surrounded by tumor tissue extending into the soft tissue. (d) Sagittal CT reformatted after the macroscopy section (Figure 3(c)) shows endomedullary cement and holes due to previous screw tracts, surrounded by denosumab changes (asterisk). Posterior soft tissue mass is noted with osteoid matrix formation suspicious for an osteosarcoma (dotted arrows). (e) Histology at the time of resection; low power view displaying highly cellular spindle cell proliferation with areas of tumor necrosis (left) and the formation of tumor osteoid (right). Scale bar of 50 *μ*m. (f) Tumor osteoid in high-power field fitting with the histological features of an osteosarcoma. Scale bar 50 *μ*m.

An X-ray at the time of initial presentation showed an osteolytic lesion in the distal femur meta-epiphysis complicated by an intra-articular pathological fracture, for which external fixation was performed (Figure 2(a)). Cortical scalloping was present on CT and no matrix formation was noted (Figures 2(b) and 2(c)). MRI showed a high signal intensity on T1-weighted images, partly due to hemorrhage after the fracture. T2-weighted images showed a heterogeneous mass with mostly high signal intensity. A posterior soft tissue mass was found and intra-articular extension of the tumor was present anterolaterally (MR images not shown, due to poor quality). Biopsy confirmed the diagnosis of conventional GCTB (Figures 2(d)–2(f)). The patient was treated with curettage and cement and the fracture was fixated with plate osteosynthesis. On a follow-up CT of the knee performed 1 year later, new osteolysis was present medial of the cement with cortex destruction, interpreted as a local recurrence (Figure 3(a)). The patient was therefore started on denosumab treatment. After 10 months on denosumab, the CT showed progression into a large medial soft tissue mass which was covered by irregular neocortex. The bone density of the endomedullary component of the local recurrence was increased most likely due to denosumab. However, within the increased soft tissue mass there was new osteoid matrix formation proximally, suspicious of malignant transformation in GCTB (Figure 3(b)). En bloc resection was performed, the macroscopic resection specimen (Figure 3(c)) and preoperative sagittal CT reformatted image (Figure 3(d)) showed corresponding endomedullary fibro-osseous matrix formation (asterisk) with osteoid deposition in a posteromedial new mass lesion. Histology at the time of en bloc resection revealed a sarcomatous appearing cellular spindle cell proliferation with areas of tumor necrosis and the formation of tumor osteoid. Histological features were in keeping with a high-grade osteosarcoma (Figures 3(e) and 3(f)). The endomedullary osteoid deposition was more regular in appearance and the criteria for sarcomatous progression were not met. Therefore this fitted with denosumab induced changes.

## 4. Discussion

Six cases of secondary mGCTB are reported containing imaging features suspicious of sarcomatous transformation in three cases. One case showed a mixed response on denosumab treatment, and the remaining two showed findings in keeping with a local recurrence. Key imaging findings suspicious of malignant transformation related to denosumab therapy are the absence of fibro-osseous matrix formation and neocortex formation on CT, and stable or increased size of the soft tissue component on CT or MRI while on denosumab treatment.

Regardless of the association with denosumab, findings that should alert the radiologist to think of malignant transformation in GCTB are areas of new cortex destruction, a new soft tissue mass, and new tumor localisations i.e., (nonpulmonary) metastases.

X-ray findings suspicious for secondary mGCTB were described previously, including less distinct margins and a soft tissue mass (present in 75% of cases), and cortical breakthrough (in 83% of cases) [14] and confirmed by Domovitov and Healey in 2010 [15]. Grading a tumor as Campanacci grade III on X-ray does not differentiate between aggressive conventional versus mGCTB [17, 24]. In both studies no CT or MRI features were described, and denosumab treatment was not taken into account [14, 15].

Secondary mGCTB may present with two distinct tumor components on CT: a previous case-report showed a low-density component which histologically corresponded to a high-grade sarcoma and a high-density component which corresponded to areas of conventional GCTB with changes related to denosumab treatment. The lag time was 13 months [21]. Our case 3 differed in that the component with sarcomatous transformation was not osteolytic but showed osteoid matrix formation, adjacent to the expected denosumab changes in the conventional GCTB component.

Tsukamoto et al. presented a case of secondary mGCTB with a lag time of six months after the start of denosumab therapy. A CT scan performed at that time revealed enlargement of a poorly defined osteoblastic mass, and a biopsy-confirmed high-grade sarcoma [18].

In summary, growth instead of a decrease in the size of the tumor during denosumab treatment is a sign pointing towards malignant transformation and this can present either as an osteolytic or osteoblastic lesion.

The mean lag time between the start of denosumab treatment and malignant transformation was 7 months (range 2–11 months). This is in accordance with previously published cases, with a lag time ranging from 6 to 13 months [17, 18, 22]. In our case series, there was no clinically relevant difference in mean lag time between patients treated with denosumab only versus patients treated with both surgery and denosumab.

The one case with surgery as the only risk factor for malignant transformation had undergone curettage with bone grafting and the latency time to mGCTB was 9 months. A hypothesis to explain surgery as the only risk factor for mGCTB may be the application of (cancellous) bone grafting, as the borders of the dead bone could form the nidus of a malignant tumor. This mechanism has been proposed for sarcomas related to bone infarction [14, 25].

The case with radiation-associated mGCTB had a lag time of 13 years, in agreement with the literature, where radiotherapy-associated mGCTB was shown to have a longer lag time than denosumab-associated sarcomatous transformation, on average, eight years [14, 16, 26, 27].

In our case series five out of six cases were women, the greater percentage of women affected with secondary mGCTB is in accordance with previous publications [15, 26], but a male predominance in secondary mGCTB has been found by others [14, 24].

In four out of six cases, the H3G34W mutation was present at the time of diagnosis of secondary mGCTB. In one case the mutation was lost (case 3), and there was one case with missing data at the time of malignant transformation (case 4). As the H3G34W mutation may be retained or lost in secondary mGCTB [20], it is key to re-evaluate the tissue from the time of diagnosis of conventional GCTB to prevent misclassification.

The misdiagnosis of primary mGCTB as conventional GCTB is an important phenomenon, which has been addressed in several recent publications [24, 28]. Therefore, in our case series on secondary mGCTB, two experienced bone tumor pathologists reviewed all available biopsies, curettages, and resection specimens to verify the primary presentation of conventional GCTB and confirm the diagnosis in all six cases. Nevertheless, we cannot completely rule out sampling error in cases only biopsy material was present at the time of first diagnosis, which may have been a cause of misclassification in our cases. Furthermore, in case 3, no biopsy was performed at the time of local recurrence. Denosumab treatment was started and mGCTB was proven after 10 months of medical treatment at the time of resection. Therefore, we cannot prove an association with denosumab in this case of secondary mGCTB.

A limitation of this case series is that imaging was done in multiple centers and not performed with standardized MRI bone tumor scanning protocols including diffusion weighted imaging and dynamic contrast enhanced sequences. Unfortunately, none of the cases in our series had MR perfusion imaging done on multiple time points. We did not have multiple timepoints of PET-CTs available to assess SUV^max^ over time on denosumab treatment. It is known that absent decrease in SUV^max^ on PET-CT is suspicious for malignant transformation of GCTB [5, 23].

## 5. Conclusions

In only 50% of cases, radiological findings were indicative of malignant transformation, even when assessed retrospectively together with histopathology. In five out of six patients presented in this case series, malignant transformation (secondary mGCTB) occurred within the first year after the start of denosumab treatment or surgery. These findings stress the importance of close clinical and imaging follow-up in the first months after denosumab therapy for GCTB, as mGCTB tends to have rapid aggressive behavior, similar to other high-grade sarcomas. The definitive diagnosis is based on histology as radiology is not sufficient.

The medical oncologist and the radiologist play an important role in the surveillance of these complex cases. On denosumab treatment, the absence of the expected pain relief in the first months after starting treatment or even new or increased pain are concerning and warrant further or new diagnostic evaluation of the tumor [13, 28]. In addition to that, if the known imaging response to denosumab treatment does not occur after 8–12 weeks, we recommend short interval follow-up (for example, repeat CT after 4 weeks) and in case of no response, biopsy needs to be repeated.

## Figures and Tables

**Table 1:** Overview of mGCTB cases: histology and therapy before malignant transformation.

Case nr	Sex (M/F)	Age at diagnosis (years)	History of malignancy Y/N	Tumor site	Histology at biopsy	Histology at the time of transformation	Time first diagnosis to malignant transformation	Previous surgery before malignant transformation Y/N	Previous radiotherapy Y/N	Denosumab Y/N	Duration of denosumab	Interval start denosumab to malignant transformation	Primary or secondary malignant transformation	Clinical outcome
1	F	54	Y, breast cancer	Left ilium	GCTB, H3G34W+	High-grade osteosarcoma, H3G34W+	12 months	N	N	Y	7 months	11 months	Secondary, denosumab associated	Died 7 months after malignant diagnosis (cause unknown), lost to follow-up

2	F	35	N	L2	GCTB, H3G34W+	Metastatic lesion in Th2: Sarcoma NOS, H3G34W+	92 months (from first diagnosis to development of metastasis with malignant transformation in Th2)	Y	Y	Y	5 months	5 months	Secondary, denosumab and surgery associated	Died 12 months after malignant diagnosis due to multiple vertebral metastases with cervicothoracic myelum compression

3	F	55	N	Left femur	GCTB, H3G34W+	High-grade osteosarcoma, H3G34W−	23 months	Y (curettage with cement)	N	Y	10 months	10 months	Secondary, denosumab and surgery associated	Died 44 months after malignant diagnosis due to pulmonary metastases

4	F	37	N	Left tarsal navicular	GCTB, H3G34W +	Recurrence with malignancy in GCTB (also areas with conventional GCTB). H3G34W missing	12 months	Y (curettage, liquid nitrogen and cancellous bone graft)	N	N	NA	NA	Secondary, postsurgery	NED

5	M	45	N	Sacrum	GCTB, H3G34W +	Highly suspicious for progression to malignant GCTB, H3G34W+	2 months	N	N	Y	2 months, neoadjuvant to surgery	2 months	Secondary, denosumab associated	NED

6	F	27	N	Right femur	GCTB, H3G34W missing (before 2000)	Resection: low-grade sarcoma, radiation associated. H3G34W+ Amputation (2 yrs later): high-grade sarcoma, H3G34W+ En bloc resection of soft tissue recurrence in the stump (4 yrs later): high-grade sarcoma (possibly radiation associated), however H3G34W− Intralesional resection of solitary L4 sarcoma metastasis (4.5 yrs later): H3G34W+	21 years	Y (multiple curettages; liquid nitrogen, cancellous bone graft and cement)	Y	N	NA	NA	Secondary, radiotherapy associated (interval 13 years)	Residual metastasis present in L4, otherwise NED

**Table 2:** Radiological characteristics of GCTB before and after malignant transformation.

Case nr	Tumor site	X ray/(PET) CT at time of diagnosis	MRI at time of diagnosis	X ray/CT at time of transformation	MRI at time of transformation
1	Left ilium	X-ray: lytic lesion with cortex destruction	Thick walled lesion with central necrosis, rim enhancement, and adjacent bone marrow edema	CT after 5 and 8 months of denosumab: absent fibro-osseous matrix and neocortex formation. Unchanged tumor size	Not performed

2	L2	X-ray: lytic lesion with cortex destruction and soft tissue mass	High T2 signal intensity with some foci of low signal	CT: recurrence with right paravertebral soft tissue mass at Th2. No osteolysis of the vertebral body	T2 hyperintense mass in Th2 with large soft tissue component, epidural extension and myelum compression, homogeneous enhancement postcontrast

3	Left femur	X-ray: lytic lesion with pathological fracture in the meta-epiphysis. CT showed cortical scalloping and focal cortex destruction	Lesion with high SI on T1 (partly due to hemorrhage after fracture) and T2, heterogeneous. Rim enhancement after contrast	CT of the local recurrence after denosumab showed new cortex destruction with a soft tissue mass, endomedullary irregular sclerosis and new osteoid matrix formation proximally	Not performed

4	Left tarsal navicular	X-ray: lytic lesion in the tarsal navicular bone without cortex destruction	Lytic lesion with cortex destruction. Iso-intense on T1, heterogeneous low signal intensity on T2. Extension into lateral cuneiform and cuboid bone	X-ray: resorption of the cancellous bone graft	MRI 3.5 months postcurettage: recurrence around cancellous bone graft, higher signal intensity lesion on T2. Extension into the talocalcaneal joint space. MRI 7.5 months postcurettage: increase of the recurrence. Extending into tarsalia and calcaneum. High T2 signal (marked increase of signal intensity compared to initial MRI at presentation). Multilobulated appearance. Bone marrow edema+

5	Sacrum	(PET)-CT: osteolysis. SUV^max^ 21	Lytic lesion with destruction of cortex, large presacral soft tissue mass. Central cystic/necrotic component high on T2, not enhancing. Low foci on T2	CT: foci of ossification in the presacral component (on denosumab). Neocortex formation+ No decrease in tumor size	Not performed

6	Right femur	X-ray showed a well demarcated lytic lesion in the lateral femoral condyle with a pathological fracture	Not performed	CT: osteolysis adjacent to the cement in the femur and cortex destruction	MRI at the time of first malignant transformation: multifocal recurrence around cement/cancellous bone graft in femur and tibia MRI 2 yrs later: large heterogeneous soft tissue mass in the popliteal fossa with fast enhancement after contrast

**Table 3:** Overview of radiological characteristics suggestive of GCTB malignant transformation, compared to baseline GCTB diagnosis.

Imaging modality	X-ray	(PET) CT	MRI
	Absent fibro-osseous matrix formation^*∗*^	No increased density on CT (HU)^*∗*^ [23]	

	Absent neocortex formation^*∗*^	Absent neocortex formation^*∗*^	
		No decrease in SUV^max^^*∗*^ [5]	
		Stable size or increase in size of the soft tissue component^*∗*^	Stable size or increase in size of the soft tissue component^*∗*^

	New cortex destruction	New cortex destruction	New cortex destruction
		New soft tissue mass	New soft tissue mass
		Metastasis (new tumor localisation)	Metastasis (new tumor localisation)

^*∗*^Specific to denosumab treatment.

## Data Availability

The radiological and histopathological data used to support the findings of this study are included within the article.
